# X-Linked thrombocytopenia causing mutations in WASP (L46P and A47D) impair T cell chemotaxis

**DOI:** 10.1186/s12929-014-0091-1

**Published:** 2014-09-09

**Authors:** Neeraj Jain, Jun Hou Tan, Shijin Feng, Bhawana George, Thirumaran Thanabalu

**Affiliations:** School of Biological Sciences, Nanyang Technological University, 60 Nanyang Drive, Singapore, 637551 Singapore

**Keywords:** Cell migration, Actin cytoskeleton, Proteosome, Cell polarity, Hematopoietic cell kinase

## Abstract

**Background:**

Mutation in the Wiskott-Aldrich syndrome Protein (WASP) causes Wiskott-Aldrich syndrome (WAS), X-linked thrombocytopenia (XLT) and X-linked congenital neutropenia (XLN). The majority of missense mutations causing WAS and XLT are found in the WH1 (WASP Homology) domain of WASP, known to mediate interaction with WIP (WASP Interacting Protein) and CIB1 (Calcium and Integrin Binding).

**Results:**

We analyzed two WASP missense mutants (L46P and A47D) causing XLT for their effects on T cell chemotaxis. Both mutants, WASP_R_^L46P^ and WASP_R_^A47D^ (S1-WASP shRNA resistant) expressed well in Jurkat^WASP-KD^ T cells (WASP knockdown), however expression of these two mutants did not rescue the chemotaxis defect of Jurkat^WASP-KD^ T cells towards SDF-1α. In addition Jurkat^WASP-KD^ T cells expressing these two WASP mutants were found to be defective in T cell polarization when stimulated with SDF-1α. WASP exists in a closed conformation in the presence of WIP, however both the mutants (WASP_R_^L46P^ and WASP_R_^A47D^) were found to be in an open conformation as determined in the bi-molecular complementation assay. WASP protein undergoes proteolysis upon phosphorylation and this turnover of WASP is critical for T cell migration. Both the WASP mutants were found to be stable and have reduced tyrosine phosphorylation after stimulation with SDF-1α.

**Conclusion:**

Thus our data suggest that missense mutations WASP_R_^L46P^ or WASP_R_^A47D^ affect the activity of WASP in T cell chemotaxis probably by affecting the turnover of the protein.

**Electronic supplementary material:**

The online version of this article (doi:10.1186/s12929-014-0091-1) contains supplementary material, which is available to authorized users.

## Background

Wiskott-Aldrich Syndrome (WAS) is a rare X-linked recessive disorder characterized by eczema, thrombocytopenia (low platelet count, small size platelets), recurrent infections, autoimmune disorders and malignancies [1,2]. It was first described in two brothers with chronic bloody diarrhea [3] and subsequently the X-linked pattern of inheritance and platelet abnormalities were characterized [4]. The gene affected in Wiskott Aldrich Syndrome was mapped to chromosome region Xp11.23 and identified by positional cloning [5]. The disease results from the mutations in a 502 amino acid protein known as Wiskott Aldrich Syndrome protein (WASP). Expression of WASP is restricted to hematopoietic cells and it plays a key role in actin cytoskeleton reorganization [6].

WASP is a proline-rich adaptor with a WH1 (WASP homology) domain at the N-terminal which mediates interaction with WIP (WASP interacting domain) [7]; the C-terminal VCA (Verprolin Central Acidic) domain stimulates the actin nucleation activity of the Arp2/3 complex [8]; the proline-rich domain before the VCA domain mediates interaction with a number of SH3 domain containing proteins [9,10] and the GBD (GTPase binding domain) mediates interaction with activated Cdc42 (Rho family of GTPases) and relieves the auto-inhibition [11]. Mutations in WASP gene can give rise to three different clinical phenotypes; classical WAS, X-linked thrombocytopenia (XLT) and X-linked severe congenital neutropenia (XLN). In the case of classical Wiskott Aldrich syndrome, WASP expression is absent [12] and most of the patients die by 10 years of age due to internal hemorrhages or recurrent bacterial or viral infections [13]. Missense mutations resulting in reduced expression of defective WASP leads to X-linked thrombocytopenia (XLT) which is characterized by thrombocytopenia with small sized platelets without immunodeficiency or eczema [14]. X-linked neutropenia (XLN) is caused by missense mutations in the GBD of WASP (L270P, S272P, I294T) which destroy the auto-inhibitory conformation of WASP and enhanced its actin polymerization activity [15,16].

A large number of point mutations in the *WASP* gene have been identified from patients with different degrees of severity [17], but the molecular mechanisms causing the disease have not been characterized for most of the mutations. More than 80% of the missense mutations are located in the WH1 domain of WASP [10] and some abolished WASP-WIP interactions [18,19] which cause instability of WASP as WIP is a chaperone for WASP [20]. It is still not clear how the majority of the missense mutations in the WH1 domain of WASP cause the disease. Out of 52 WASP missense mutations reported, twelve of the mutations are outside the WH1 domain and do not impair the ability to suppress the growth defect of *las17Δ* yeast strain suggesting that regulation of actin dynamic by WASP is unaffected [18]. Our laboratory has carried out a more comprehensive study of all 40 mutations present in the WH1 domain of WASP and found that only 13 point mutations out of 40 abolished formation of a functional WASP-WIP complex in *S. cerevisiae las17Δ* strain [18]. Thus, it is still not clear how the majority of the remaining point mutations cause the disease. The WH1 domain of WASP has also been shown to interact with CIB1 (Calcium and Integrin Binding) and this interaction is critical for adhesion of platelets to fibrinogen [21]. CIB1 is a 22 kDa EF-hand containing protein which is expressed ubiquitously and identified as a binding partner of the cytoplasmic tail of platelet integrin αIIbβ3 [22].

In this study, we have characterized two WASP missense mutations L46P and A47D in the WH1 domain causing XLT. These two missense WASP mutants were found to express well in Jurkat^WASP-KD^ T cells at levels comparable to that of wild type WASP; however, expression of WASP_R_^L46P^ or WASP_R_^A47D^ did not rescue the chemotaxis defect of Jurkat^WASP-KD^ T cells and the Jurkat^WASP-KD^ T cells expressing the mutants displayed an abnormal actin cytoskeleton and defective polarization after stimulation with SDF-1α. While WASP exists in a closed conformation in the presence of WIP, both WASP^L46P^ and WASP^A47D^ mutants were found to be in open conformation in the presence of WIP. While phosphorylation of tyrosine residue promotes proteolytic degradation of wild type WASP, both mutants were relatively stable and had reduced tyrosine phosphorylation after SDF-1α stimulation. Our results suggest that these that mutations affect WASP turnover resulting in defective chemotaxis.

## Methods

### Cell culture

Phoenix Amphotropic packaging cell line (ATCC, USA) was maintained in DMEM/10% FBS at 37°C while Jurkat (clone E6-1) (ATCC, USA) were maintained in RPMI/10% FBS at 37°C. Jurkat^WASP-KD^ T cells were generated by transducing Jurkat T cells with retrovirus (generated using amphotropic packaging cells) expressing human WASP specific shRNA (S1-WASP shRNA) (GCAGGGAATTCAGCTGAACAA) under the transcriptional control of the U6 promoter and GFP under the CMV promoter. The infected cells were FACS sorted using GFP fluorescence. We generated shRNA resistant WASP (WASP_R_) mutants by introducing 4 silent mutations (GCA*A*GG*T*AT*C*CA*A*CTGAACAA) in the region targeted by the shRNA. WASP_R_ or its mutants (tagged with RFP at their C-terminus) were cloned in an EBV based plasmid and expressed under the transcriptional regulation of the *WASP* promoter. For expression of WASP or its mutants in HEK293T cells, the gene was cloned under the transcriptional regulation of the CMV promoter in the pFIVcopGFP plasmid (System Biosciences).

### Generation of stable cell lines

Jurkat^WASP-KD^ T cells were microporated with plasmid expressing WASP_R_-RFP (WT or mutants) using the Neon transfection system (Invitrogen, CA, USA) according to manufacturer’s instructions. In brief, 5 x 10^6^ Jurkat^WASP-KD^ T cells for each transfection were washed with PBS, resuspended in 100 μl of resuspension buffer (R-buffer) and mixed with 10 μg of plasmid DNA. The cells-DNA mixture was subjected to three pulses with pulse width 10 ms at 1500 V. Transfected cells after microporation were selected with neomycin (G418, P02-012) (PAA Laboratories, Pasching, Austria) for one week (1.5 mg/ml) before analysis. Medium containing G418 was changed once every 2 to 3 days for a week until the exogenous WASP was stably expressed in the cells.

### Chemotaxis assay using Dunn chamber

Sterilized coverslips were coated with 2 μg/ml fibronectin in PBS for 1 hour at 37°C in a CO_2_ incubator before adding 2.25 X 10^6^, Jurkat T cells. The outer and inner well of Dunn chamber (Hawksley & Sons Ltd, UK) [23] were filled with complete RPMI media. The coverslip with attached cells was then inverted onto the Dunn chamber and media in the outer well was replaced with complete RPMI media containing chemokine (5nM SDF-1α) (Pepro Tech, London, UK). The coverslip was sealed using a mixture of hot wax. Migrating cells on the annular bridge were imaged using an inverted microscope (Olympus IX81) with a 10X objective lens. Time-lapsed images were digitally captured every 2 minutes for a period of 3 hours using a CoolSNAP^HQ^ camera. Migration paths of the cells were traced and analyzed from the series of images using Metamorph software (Molecular devices, CA, USA). Twenty cells were analyzed for each set of experiment and each experiment was repeated thrice.

### Transwell migration assay

Jurkat^WASP-KD^ T cells expressing various WASP_R_ mutants were serum starved for two hours and 2 x 10^5^ cells in 100 μl RPMI were added to the upper chamber of a transwell (5.0 μm pore size) (Costar, Cambridge, MA). The lower well was filled with 600 μl containing 100 ng/ml of SDF-1α and incubated at 37°C for 3 hours in CO_2_ incubator. Cells in the lower well were counted using a hemocytometer. The percentage of cells migrated was calculated as the ratio of the number of cells detected in the lower well over the total cell number.

### Immunofluorescence and BiFC

Jurkat T cells or Jurkat^WASP-KD^ T cells expressing WASP_R_ or mutants were resuspended in RPMI-1640 media containing 25 mM HEPES (pH 7.4) and incubated for 20 min at 37°C, CO_2_ incubator. Cells after incubation were plated on poly-L-lysine coated coverslips and allowed to attach for 10 min. Attached cells were stimulated with SDF-1α (10 nM) for 5 min and fixed (4% formaldehyde/PBS) for 20 min. The fixed cells were permeabilized (0.2% Triton X-100/PBS) for 30 min, blocked with 1% BSA/PBS, washed three times and stained with Alexa594 phalloidin (Molecular probes, Eugene, Oregon, USA). Fluorescent images were acquired using an Olympus microscope fitted with Photometrics CoolSNAP^HQ2^ camera. The experiment was repeated at least three times and 40 cells were evaluated for each experiment.

Bi-molecular Fluorescence Complementation (BiFC) was carried out as described previously [24,25]. In brief, a WASP (WT or mutants) sensor construct was co-transformed with WIP in an *S. cerevisiae* yeast strain. The transformed yeast cells were visualized for fluorescence using an Olympus microscope with CoolSNAP^HQ^ camera (Roper scientific) and fluorescence intensity was calculated using Metamorph software. Similar experiments were performed in mammalian HEK293T cells. In brief, HEK293T cells were co-transfected with WIP-His and WASP (WT or mutants) sensors. After 36 hours of transfection, the cells were visualized and the fluorescence intensity of the transfected cells was quantified using Metamorph software.

### Proteasome inhibition study and immunoblotting

WASP_R_-RFP (WT or mutants) expressing Jurkat T cells were incubated with proteasome inhibitor MG132 (50 μM) (Enzo Life Sciences, Plymouth Meeting, PA, USA) or calpain inhibitor Calpeptin (50 μg/ml) (Calbiochem, San Diego, CA, USA) for 5 h. After incubation, cells were lysed using RIPA lysis buffer, the resulting lysate (30 μg of protein) was boiled in a SDS-PAGE sample buffer, resolved on a 10% SDS-PAGE gel and transferred onto a nitrocellulose membrane. The membrane was probed with primary antibody (WASP D1, Santa Cruz Biotechnology, CA, USA) followed by secondary antibody conjugated with horse radish peroxidase (HRP). Anti-GAPDH (Ambion, Austin, TX, USA) was used as a loading control. *S. cerevisiae* cell lysis was performed as described [18]. In brief, yeast cells were harvested by centrifugation and the pellet resuspended in 1.85 N NaOH/1.0 M β-mercaptoethanol. The protein in the lysate was precipitated using 20% trichloroacetic acid (TCA) and protein pellet was washed once with cold acetone. The protein was resuspended in SDS page loading buffer and analysed with appropriate primary antibodies.

For analysis of WASP phosphorylation after activation with chemokine SDF-1α, Jurkat T cells expressing WASP or His-tagged mutants were serum-starved overnight followed by activation with SDF-1α (100nM) for 5 min. Cells were lysed immediately and His-tag pull down was performed. Pull down samples were analysed by western blot using anti-His (WASP) (Delta biolabs, Gilroy, CA, USA) and anti-phosphotyrosine (4G10; Upstate Biotechnology, Lake Placid, NY) antibodies. In addition, WASP or His-tagged mutants were expressed together with activated Cdc42 (Cdc42^G12V^) in HEK293T cells. Thirty-six hours post-transfection, the cell lysate was prepared and a His-tag pull down assay was performed. The tyrosine phosphorylation status of the WT and mutant WASP was assessed by western blot using the 4G10 antibody.

### Statistical analysis

Statistical significance analysis was performed using unpaired student’s t-test and *p* < 0.05 was considered statistically significant. Values presented in bar charts represent the mean ± S.D for at least three independent experiments.

## Results

### Expression of WASP_R_^L46P^ and WASP_R_^A47D^ does not rescue the chemotactic response of Jurkat^WASP-KD^ T cells to SDF-1α

A large numbers of missense mutations in the WH1 domain of WASP have been linked to Wiskott Aldrich Syndrome and XLT [10]. WIP binds to the WH1 domain of WASP (residues 101–151) and stabilizes the closed conformation of WASP [25] and protects WASP from calpain mediated proteolysis [19,20,26]. CIB1 binds to the WHI domain of WASP (residues 1–100) and this complex is critical for αIIbβ3-mediated cell adhesion and platelet aggregation [21]. In this study, we analyzed twenty-five missense mutants in the WH1 domain of WASP for their interaction with CIB1 and found six of the WASP mutations (G40V, T45M, L46P, A47D, P58L, G70W) abolished WASP-CIB1 interaction without affecting WASP-WIP interaction (Additional file 1: Figure S1). Three of the WASP^WH1^ missense mutations have been reported to cause WAS (WASP^G40V^, WASP^P58L^ and WASP^G70W^) and the remaining three mutations (WASP^T45M^, WASP^L46P^, WASP^A47D^) have been reported to cause XLT [10]. XLT is characterized by reduced number of platelets as well as small platelets [27].

**Figure 1:**
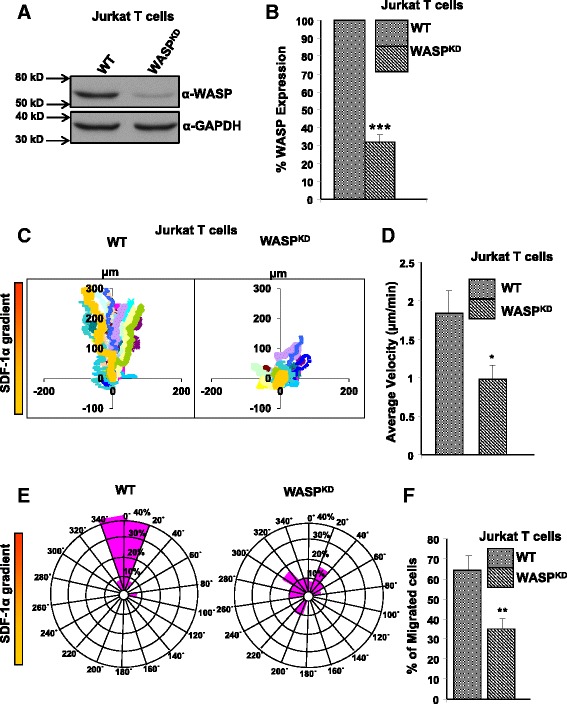
**WASP knockdown Jurkat T cells are impaired in chemotaxis towards SDF-1α. A)** Jurkat T cells were transduced with retrovirus expressing human WASP specific shRNA and EGFP. The cells were FACS sorted and the efficiency of knock down was determined by western blot using anti-WASP and anti-GAPDH antibody. **B)** WASP mRNA levels in Jurkat T cells and Jurkat^WASP-KD^ T cells were quantified by real-time PCR. ***P < 0.001 compared to WT Jurkat T cells. **C)** Vector plots of migration paths of 20 randomly chosen cells from WT Jurkat T cells or Jurkat^WASP-KD^ T cells exposed to a gradient of SDF-1α in the Dunn chamber. The starting point of each cell is at the intersection of the X and Y axes. The source of SDF-1α was at the top. **D)** Migration velocity of WT and Jurkat^WASP-KD^ T cells shown as the mean of the velocities of 60 randomly chosen cells (20 cells each from 3 sets of experiments) *p < 0.05. **E)** Circular histograms showing the percentage of cells at the final positions in each of the sectors (20°). The source of SDF-1α was at the top. **F)** Transwell migration assay was performed with WT Jurkat T cells and Jurkat^WASP-KD^ T cells. 2 X 10^5^ cells were loaded in upper well and allowed to migrate towards SDF-1α (100 ng/ml) containing media in the lower well for 3.0 hrs. Cells migrated to the lower well were counted and plotted as percentages of total cells added to the upper well. Data represent the mean of three independent experiments. **p < 0.01 compared to WT Jurkat T cells.

**Figure 2:**
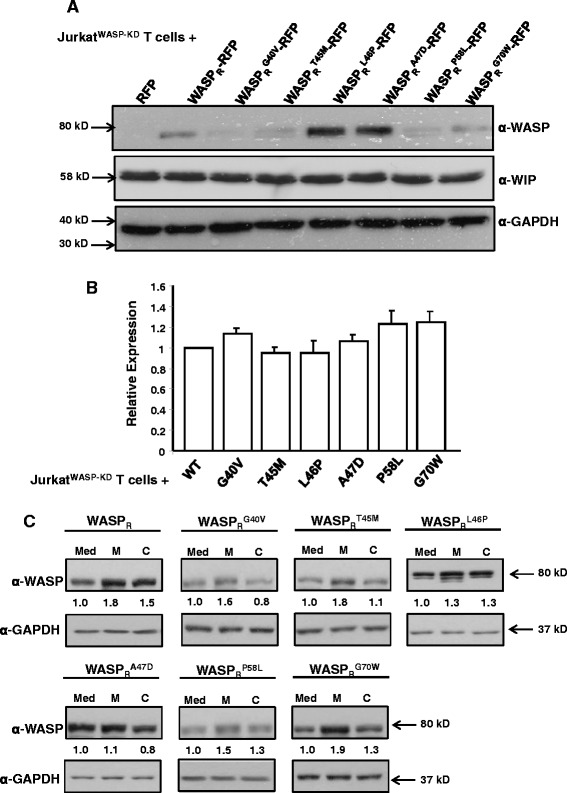
**WASP mutants WASP**_**R**_^**L46P**^ **and WASP**_**R**_^**A47D**^ **are stably expressed in Jurkat** ^**WASP-KD**^ **T cells. A)** Jurkat^WASP-KD^ T cells expressing RFP or WASP_R_-RFP fusions (WT or mutants) were generated by microporating Jurkat^WASP-KD^ T cells with plasmids expressing the constructs under the transcriptional regulation of WASP promoter followed by one week of selection with neomycin. The cell lysate were resolved and probed with either anti-WASP, anti-WIP and anti-GAPDH antibodies. **B)** Total RNA from Jurkat^WASP-KD^ T cells expressing WASP-RFP fusions or mutant derivatives were isolated and used to carryout quantitative real time PCR analysis to analyze expression of WASP or its mutant at mRNA level. **C)** Jurkat T cells expressing the RFP tagged WT or mutants WASP were incubated with either MG132 or calpeptin for 5 hours. Cells were lysed and immunoblotted for WASP and GAPDH antibodies.

In order to carry out functional assays of the six mutants in Jurkat T cells, we first generated WASP specific shRNA (S1-WASP-shRNA) to knock down the expression of endogenous WASP. WASP and N-WASP have more than 50% sequence homology [28]. We therefore tested the specificity of S1-WASP-shRNA by co-transfecting HEK293T cells with expression plasmids for S1-WASP-shRNA and either human N-WASP or WASP. We found that the S1-WASP-shRNA did not target human N-WASP (data not shown). We generated S1-WASP-shRNA expressing retrovirus by transfection of amphotropic packaging cells [29]. The virus supernatant was used to transduce Jurkat T cells using spinoculation at a transduction efficiency of 43% (data not shown). The transduced Jurkat T cells stably expressing GFP were sorted by flow cytometry (data not shown) and propagated. Western blot analysis of sorted Jurkat^WASP-KD^ T cells showed effective knockdown of endogenous WASP expression (Figure 1A). This was further validated by qPCR which showed that mRNA level of WASP in Jurkat^WASP-KD^ T cells was reduced by 68% compared to WT Jurkat T cells (Figure 1B). WASP has been shown to be critical for T cell chemotaxis [30]. Consistent with previous studies, our Jurkat^WASP-KD^ T cells were defective in their chemotactic response to the chemokine SDF-1α as assessed in Dunn chamber (Figure 1C-E). Compared to 85% of WT Jurkat T cells, only 15% of Jurkat^WASP-KD^ T cells were found in the 40° arc facing the chemokine source (Figure 1E). Moreover the migration velocity of Jurkat^WASP-KD^ T cells (0.98 μm/min) was significantly reduced compared to WT Jurkat T cells (1.84 μm/min) (Figure 1D). The defect in chemotaxis of Jurkat^WASP-KD^ T cells was further confirmed by transwell assays (Figure 1F). In order to express the WASP mutants in Jurkat^WASP-KD^ T cells, we generated WASP_R_, WASP which is resistant to the S1-WASP-shRNA by introduction 4 silent mutations at the region of WASP gene targeted by shRNA. The expression level of WASP and WASP_R_ was found to be comparable when tested in both HEK293T cells and Jurkat T cells (Additional file 2: Figure S2A, B) which suggest that the introduction of silent mutations in WASP gene does not affect the translation efficiency of the protein. The genes encoding WASP or its mutants were placed under the transcriptional regulation of the human WASP promoter. Plasmids expressing wild type WASP_R_ or its mutants (WASP_R_^G40V^, WASP_R_^T45M^, WASP_R_^L46P^, WASP_R_^A47D^, WASP_R_^P58L^ and WASP_R_^G70W^) tagged with RFP were microporated into Jurkat^WASP-KD^ T cells and selected with neomycin for one week before analyzing the WASP expression by western blotting. The expression of WASP_R_^L46P^ and WASP_R_^A47D^ can be readily detected whereas expression levels of the other four mutants (WASP_R_^G40V^, WASP_R_^T45M^, WASP_R_^P58L^ and WASP_R_^G70W^) were decreased (Figure 2A). This decrease is due neither to any defect at the transcription level (Figure 2B) nor to any changes in WIP expression levels (Figure 2A). However, all six mutants express well in HEK293T cells suggesting that the reduced expression of the four WASP mutants was restricted to T cells (Additional file 2: Figure S2C, D). WIP is known to interact with the WH1 domain of WASP and protect WASP from cytoplasmic proteases [20]. Reduced expression of these four WASP mutants in T cells could be the possible cause of the disease in patients harboring these mutations. None of the six mutations affected interaction with WIP (Additional file 1: Figure S1), indicating that instability was not due to loss of interaction with WIP. This may indicate that WIP is not the only chaperone for WASP in T cells.

The effect of proteasome (MG132) and calpain (calpeptin) inhibitors on the expression level of WASP and its mutants was examined. We found that pre-incubation of Jurkat T cells expressing the WASP_R_ WT and mutants (G40V, T45M, P58L and G70W) with MG132 resulted in significant increase in WASP expression level (WASP_R_, 1.8 ± 0.19 fold; G40V, 1.6 ± 0.30 fold; T45M, 1.8 ± 0.40 fold; P58L, 1.5 ± 0.02 fold; G70W, 1.9 ± 0.30 fold) (mean of three experiments). However, there was no significant increase in mutants (L46P and A47D) expression level observed after MG132 treatment (L46P, 1.34 ± 0.50 fold; A47D, 1.1 ± 0.15 fold). Pre-incubation of Jurkat T cells expressing all six WASP mutants with calpeptin did not result in an increase in expression compared to significant increase in WT WASP expression. Thus all together results suggest that four WASP mutants (G40V, T45M, P58L and G70W) are unstable in Jurkat T cells due to their proteasome mediated degradation.

**Figure 3:**
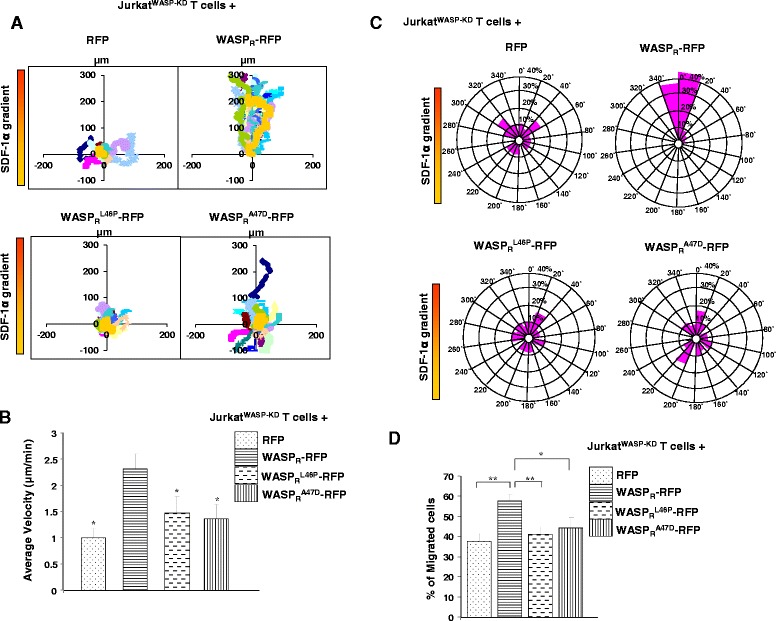
**Expression of WASP** ^**L46P**^ **or WASP** ^**A47D**^ **does not rescue the chemotaxis defect of Jurkat** ^**WASP-KD**^ **T cells. A)** Vector plots of migration paths of 20 randomly chosen cells from Jurkat^WASP-KD^ T cells expressing RFP, WASP_R_-RFP, WASP_R_^L46P^-RFP, WASP_R_^A47D^-RFP exposed to a gradient of SDF-1α in the Dunn chamber. Time-lapse images were captured for 3 hours at 2.0 min intervals. The starting point of each cell is at the intersection of the X and Y axes. The source of SDF-1α was at the top. **B)** Migration velocity of each cell type as in panel A, mean of velocities of 60 randomly chosen cells (20 cells each from 3 sets of experiments) * *P < 0.05* compared to Jurkat^WASP-KD^ T cells expressing WASP_R_-RFP. **C)** Circular histograms showing percentage of cells at their final positions in each of the sectors (20°). The source of SDF-1α was at the top. **D)** In transwell migration assay, cells were loaded in upper well and allowed to migrate towards SDF-1α (100 ng/ml) containing media in the lower well for 3.0 hours. Cells migrated to the lower well were counted and the percentage of cells migrated to the lower well was calculated. Data represent the mean of three independent experiments. * P < 0.05, ** P < 0.01.

The two mutants (WASP_R_^L46P^, WASP_R_^A47D^) which are stable and readily detected in Jurkat^WASP-KD^ T cells were analyzed for their ability to rescue chemotaxis using a Dunn chamber assay. As shown in vector plot, expression of WASP_R_^L46P^-RFP or WASP_R_^A47D^-RFP failed to rescue the chemotactic defect of Jurkat^WASP-KD^ T cells, unlike the WASP_R_-RFP (Figure 3A). Quantitative analysis revealed that the migration speed of Jurkat^WASP-KD^ T cells expressing WASP_R_^L46P^-RFP (1.46 μm/min) or WASP_R_^A47D^-RFP (1.36 μm/min) was significantly reduced compared to Jurkat^WASP-KD^ T cells expressing the WASP_R_-RFP (2.3 μm/min) (Figure 3B). Circular histogram showing the overall directionality of cell migration revealed that 86.6% of Jurkat^WASP-KD^ T cells expressing WASP_R_-RFP moved to a position within the 40° of arc facing the chemokine source. By contrast only 21.32% and 20.6% of Jurkat^WASP-KD^ T cells expressing WASP_R_^L46P^-RFP or WASP_R_^A47D^-RFP, respectively moved to this position, comparable to control urkat^WASP-KD^ T cells expressing RFP alone (10% of total). Thus, the WASP_R_^L46P^ or WASP_R_^LA47D^ mutants do not rescue the chemotactic defect of Jurkat^WASP-KD^ T cells (Figure 3C).

To confirm the chemotactic migration defect of Jurkat^WASP-KD^ T cells expressing WASP_R_^L46P^-RFP or WASP_R_^A47D^-RFP, a transwell migration assay was performed. We found that 41.3% of WASP_R_^L46P^-RFP or 44.4% of WASP_R_^A47D^-RFP expressing Jurkat^WASP-KD^ T cells migrated to the lower well compared to 57.5% of WASP_R_-RFP expressing cells suggesting that WASP_R_^L46P^ or WASP_R_^A47D^ mutants are unable to rescue the impaired chemotaxis of Jurkat^WASP-KD^ T cells towards SDF-1α (Figure 3D). Taken together these results suggest that the missense mutation (WASP^L46P^ or WASP^A47D^) abolished WASP function in chemotaxis.

### Abnormal actin cytoskeleton reorganization of Jurkat^WASP-KD^ T cells expressing WASP^L46P^ and WASP^A47D^ mutants in response to SDF-1α stimulation

**Figure 4:**
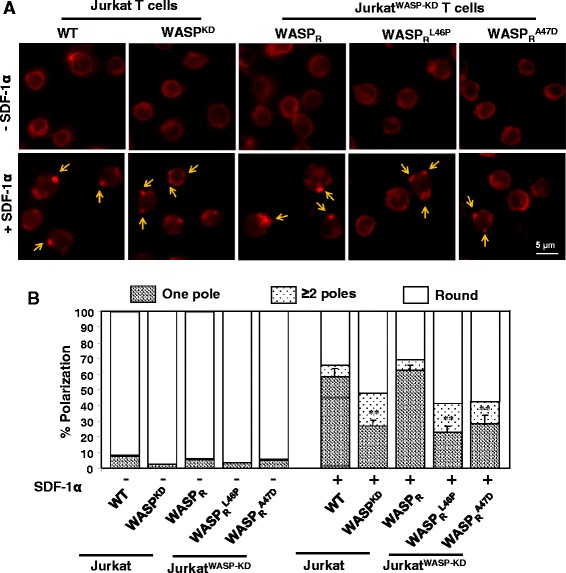
**Jurkat** ^**WASP-KD**^ **T cells expressing WASP mutants exhibit abnormal actin cytoskeleton reorganization upon SDF-1α stimulation. A)** WT or Jurkat^WASP-KD^ T cells expressing WASP_R_ or mutants were plated on poly-L-lysine coated coverslip, stimulated for 5 min with SDF-1α, fixed and stained with Alexa 594 phalloidin. Arrows indicate the actin patches. **B)** Quantification of actin polarization from three independent experiments as described in materials and methods. **p < 0.01 compared to WT Jurkat T cells.

The actin cytoskeleton plays a critical role in chemotaxis and WASP regulates the actin cytoskeleton [31] and results from previous section suggests that expression of either of the WASP missense mutants (WASP_R_^L46P^ or WASP_R_^A47D^) did not rescue the chemotaxis defect of Jurkat^WASP-KD^ T cells towards chemokine SDF-1α (Figure 3). SDF-1α has been shown to regulate actin cytoskeleton reorganization and cell polarization [32]. In order to investigate the reorganization of the actin cytoskeleton in response to SDF-1α, Jurkat T cells or Jurkat^WASP-KD^ T cells expressing mutants (WASP_R_^L46P^ or WASP_R_^A47D^) were stimulated with SDF-1α for 5 min, fixed and stained with phalloidin. Unstimulated cells, WT Jurkat T cells or Jurkat^WASP-KD^ T cells expressing WASP or its mutants exhibited a ring of actin at the cell periphery. Upon stimulation with SDF-1α, more than 50% of the Jurkat T cells or Jurkat^WASP-KD^ T cells expressing WASP_R_ lost their rounded morphology and exhibited a polarized actin cytoskeleton with one prominent patch of actin (Figure 4A). In contrast Jurkat^WASP-KD^ T cells or Jurkat^WASP-KD^ T cells expressing mutants had a reduced percentage of cells (~25 to 30%) with actin polarized to one pole and most of them (~55%) retained a rounded shape. A fraction (>20%) of Jurkat^WASP-KD^ T cells or Jurkat^WASP-KD^ T cells expressing WASP mutants exhibited abnormal polarization with multiple patches of actin after stimulation with SDF-1α while less than 10% of Jurkat T cells or Jurkat^WASP-KD^ T cells expressing WASP_R_ had more than one pole after stimulation (Figure 4B). This suggests that the abnormal actin cytoskeleton organization could account for the observed chemotactic defect of Jurkat^WASP-KD^ T cells expressing WASP mutants.

### WASP mutants (WASP^L46P^ and WASP^A47D^) exists in open conformation

**Figure 5:**
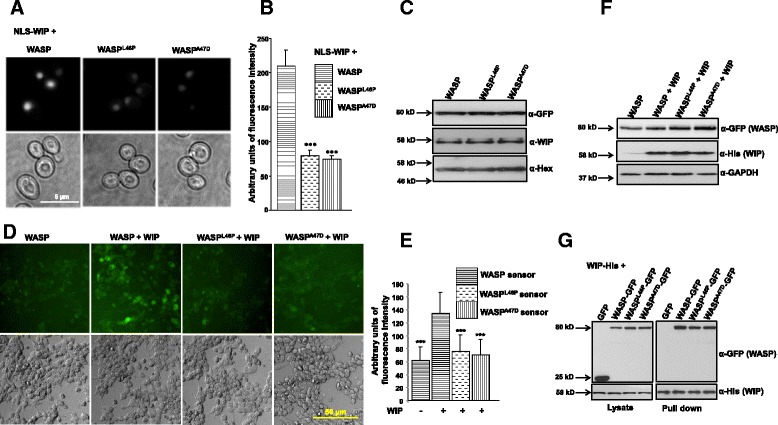
**WASP mutants exist in open conformation even in the presence of WIP. A)** *S. cerevisiae* cells expressing NLS-WASP-Sensor or mutant (L46P, A47D) sensors + NLS-WIP were grown to exponential phase at 24°C and analyzed by fluorescence microscopy. **B)** Quantification of fluorescence intensity of *S. cerevisiae* cells expressing NLS-WASP-Sensor or mutants (L46P, A47D) + NLS-WIP. ***p < 0.001 compared to NLS-WASP + NLS-WIP expressing cells. **C)** Western blot analysis of *S. cerevisiae* cells expressing of NLS-WASP-Sensor or mutants + NLS-WIP using anti-GFP, anti-WIP and anti-Hexokinase serum. **D)** HEK293T cells were co-transfected with WASP or mutants (L46P, A47D) sensor with WIP and analyzed by fluorescence microscopy. **E)** Quantification of fluorescence intensity of WASP or mutants (L46P, A47D) in the presence of WIP. ***p < 0.001 compared to WASP sensor + WIP expressing cells. **F)** Western blot analysis of HEK293T cells expressing WASP or mutant sensors with WIP using anti-GFP (WASP), anti-His (WIP) and anti-GAPDH. **G)** His tag pull down assay showing the interaction of WASP or mutants (L46P, A47D) (tagged with GFP) with WIP-His in HEK293T cells.

WASP exists in two different conformations. In its resting state, WASP adopts a closed conformation that is stabilized by WIP [25]. After T cell receptor ligation, Rho family GTPases, such as Cdc42 and tyrosine kinases bind and activate WASP [9,33] by inducing a conformational change. In order to study the effect of missense mutations on the conformation of WASP, a Bi-molecular Fluorescence Complementation (BiFC) assay was performed [25]. The YFP molecule was split into two fragments; for the WASP and its mutants (WASP_R_^L46P^ or WASP_R_^A47D^) YFP_1–154_ was fused to the N-terminus while YFP_155–238_ was fused to the C-terminus to generate the WASP sensor (NLS-YFP_1–154_-WASP-YFP_155–238_) or corresponding WASP mutant sensors. The NLS localizes the sensor to the nucleus which protects it from cytoplasmic proteases [25]. It has been reported that 95% of WASP exists as a complex with WIP [34]. Thus we expressed NLS-WASP or mutant sensor together with NLS-WIP in yeast cells. The fluorescence intensity of cells was calculated as described previously [25]. We found that fluorescence intensity of both WASP mutant sensors was reduced compared to the WASP sensor in the presence of WIP (Figure 5A and B). The reduced fluorescence intensity of WASP mutant sensors in the presence of WIP was not due to their poor expression compared to WASP sensor (Figure 5C). We further analyzed the conformation of WASP and its mutants in a mammalian system. WASP or mutant sensors were co-transfected with WIP in HEK293T cells. After 36 hours of transfection, fluorescence intensity of the cells was observed and quantified. Similar to the results obtained in yeast, the fluorescence intensity of WASP was increased in the presence of WIP, suggesting that WASP exists in its closed conformation. However, the fluorescence intensity of WASP mutants was significantly reduced even in the presence of WIP (Figure 5D-F). The reduced fluorescence intensity of the mutant WASP sensors (WASP^L46P^ and WASP^A47D^) was not due to reduced expression or decreased interaction with WIP (Figure 5G). This suggests that mutations altered the WASP closed conformation.

### WASP mutants (WASP^L46P^ and WASP^A47D^) have reduced tyrosine phosphorylation after SDF-1α stimulation

**Figure 6:**
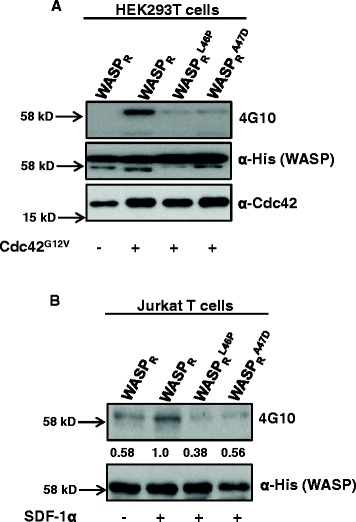
**WASP** ^**L46P**^ **and WASP** ^**A47D**^ **mutants have reduced tyrosine phosphorylation after SDF-1α stimulation. A)** HEK293T cells expressing WASP_R_ or mutants (L46P, A47D) (tagged with His tag) together with or without activated form of Cdc42 (Cdc42^G12V^). Thirty-six hours after transfection, a His-tag pull down assay was performed and analyzed by western blot for WASP (anti-His), Cdc42 and 4G10 (anti-phosphotyrosine antibody). **B)** Jurkat T cells expressing WASP_R_ or its mutants (L46P, A47D) (tagged with His tag) were stimulated with SDF-1α for 5 min and lysed. His tag pull down assay was performed and analyzed by western blot for phosphorylation of WASP or its mutants (L46P, A47D) using 4G10 antibody.

WASP has been shown to be activated by many binding partners. One of the mechanisms for WASP activation is mediated by binding of Cdc42 to the GTPase binding domain of WASP. This provokes the release of WASP from its inhibited state and contributes to subsequent WASP tyrosine phosphorylation [35-37]. In our study, co-expression of WASP with activated Cdc42 (Cdc42^G12V^) in HEK293T cells resulted in increased WASP tyrosine phosphorylation (Figure 6A), supporting previous studies whereby Cdc42 relieved the WASP auto-inhibitory conformation and increased the accessibility of WASP to cellular kinases [38-40]. Compared to WASP, phosphorylation of WASP mutants (L46P, A47D) was significantly reduced (Figure 6A). Moreover, we found that the phosphorylation of mutant WASP was significantly reduced in Jurkat T cells after stimulation with SDF-1α (Figure 6B). Previous studies have shown that SDF-1α stimulation activates Cdc42 which is required for WASP-Cdc42 interaction and thus critical for T cell chemotaxis [30]. Although the two WASP mutations (L46P, A47D) do not affect WASP-Cdc42 interaction (data not shown), these mutations affected the WASP conformation and thus its phosphorylation, which is critical for T cell chemotaxis.

## Discussion

WASP is a multi-domain proline-rich protein which regulates the actin cytoskeleton by modulating the actin nucleation activity of the Arp2/3 complex [41]. Most of the missense mutations causing WAS/XLT are found in the WH1 domain of WASP [10] and it has been suggested that loss of interaction with WIP is causal for the disease [19]. However we have previously shown that the majority of the mutations in the WH1 domain do not affect the formation of a functional WASP-WIP complex [18]. In order to characterise the molecular defect of the other mutations in the WH1 domain we screened twenty-five WASP missense mutations for interaction with CIB1 and found six WASP missense mutants (WASP^G40V^, WASP^T45M^, WASP^L46P^, WASP^A47D^, WASP^P58L^ and WASP^G70W^) that are defective in binding to CIB1, but not to WIP (Additional file 1: Figure S1). Four of the missense WASP mutants (WASP^G40V^, WASP^T45M^, WASP^P58L^, WASP^G70W^) were found to be relatively unstable in Jurkat^WASP-KD^ T cells but their expression was increased in the presence of proteasome inhibitor (Figure 2). Thus, suggest that these WASP mutations reduce stability by increasing proteasome-mediated degradation. Moreover these four WASP mutants were expressed well in HEK293T cells which suggest that the reduced expression of the four WASP mutants was T cell specific (Additional file 2: Figure S2C, D) and also indicates that WIP is not the only chaperone for WASP in T cells. The expression of the other two missense mutants (WASP^L46P^ and WASP^A47D^) were readily detected (Figure 2A).

Jurkat^WASP-KD^ T cells were defective in their chemotactic response to the chemokine SDF-1α (Figure 1C, D, E, F) and expression of WASP_R_ (shRNA resistant) but not its mutant derivatives (WASP_R_^L46P^ or WASP_R_^A47D^) rescued the chemotaxis defect (Figure 3). CIB1 has been shown to promote megakaryocyte migration towards SDF-1α [42] and expression of CIB1 has been reported to be upregulated in breast cancer tissue [43] suggesting a regulatory role for CIB1 in cell migration. Thus, the loss of interaction of WASP^L46P^ or WASP^A47D^ with CIB1 could be one of the reasons for defective chemotaxis of Jurkat^WASP-KD^ T cells expressing the WASP mutants.

Analysis of actin cytoskeleton organization after SDF-1α stimulation revealed that cells expressing either the WASP^L46P^ or WASP^A47D^ mutants were impaired in actin cytoskeleton reorganization as most of the cells exhibited a rounded shape and a significant number of cells had more than one pole (Figure 4). Polarization of T-lymphocytes in response to chemokine stimulation is essential for chemotaxis as inhibition of lymphocytes polarization by pentoxifylline inhibits their chemotactic response [44]. Thus, the defect in T cell polarization of Jurkat^WASP-KD^ T cells expressing mutants (WASP_R_^L46P^ or WASP_R_^A47D^) in the presence of chemokine SDF-1α could account for their impaired chemotaxis.

WASP exists in two conformations, an open active conformation and a closed inactive conformation which is stabilized by WIP [11,25]. Binding of activated Cdc42 and/or phosphorylation of tyrosine^291^ relieves the auto-inhibition and promote activation of the Arp2/3 complex [36]. While WASP was in a closed conformation in the presence of WIP, both WASP^L46P^ and WASP^A47D^ were found to be in an open conformation even in the presence of WIP (Figure 5) suggesting defects in the conformational regulation of WASP in these two mutants. Expression of WASP^L46P^-RFP or WASP^A47D^-RFP was elevated compared to WASP_R_-RFP expression in Jurkat^WASP-KD^ T cells suggesting that the mutations might have increased the stability of WASP. Moreover, co-expression of WASP with Hck (hematopoietic cell kinase) in HEK293T cells caused degradation of WASP, while co-expression of WASP mutants with Hck did not result in proteolytic degradation of WASP mutants (Additional file 3: Figure S3). This suggests that the WASP mutants (WASP_R_^L46P^ or WASP_R_^A47D^) are highly stable. Turnover of WASP is essential for chemotaxis as inhibition of calpain mediated WASP turnover resulted in impairment in migration of dendritic cells [45]. Compared to WT WASP, the phosphorylation of both mutants was reduced when expressed together with activated Cdc42 (Cdc42^G12V^) in HEK293T cells (Figure 6A). In addition, both the mutants had reduced tyrosine phosphorylation in Jurkat T cells activated with chemokine SDF-1α (Figure 6B). Tyrosine phosphorylation of WASP has been shown to target WASP for proteasome mediated degradation which is critical for WASP turnover [46]. The reduced tyrosine phosphorylation of WASP mutants in Jurkat T cell stimulated with SDF-1α is probably the cause of their increased stability and hence the altered chemotactic response.

## Conclusion

We have found that two WASP mutations (L46P and A47D) causing XLT affect the function of WASP in T cell chemotaxis. Jurkat^WASP-KD^ T cells expressing these mutants were found to have abnormal actin cytoskeleton reorganization upon stimulation with SDF-1α. These two mutations keep WASP in an open conformation but reduced tyrosine phosphorylation following SDF-1α stimulation leading to enhanced WASP stability. The turnover of WASP is essential for chemotaxis and these two mutations probably affect the activity of WASP in this process by enhancing the stability of WASP.

## Additional files

Additional file 1: Figure S1. Six missense mutations in the WH1 domain abolish WASP-CIB1 interaction. (A) Yeast two hybrid strains harboring Gal4 DNA-Binding domain-WASP_1–265_ (WT or its mutants) and Gal4 Activation Domain-CIB1 fusion were streaked on selective plates (−T-L: minus Tryptophan/Leucine or –T-L-H: minus Tryptophan/Leucine/Histidine). The plates were incubated at 30°C and photographed after 5 days. (B) Mutations affecting WASP-CIB1 interaction do not affect WASP-WIP complex formation**.** *S. cerevisiae* strain PJ69-4A was co-transformed with plasmids expressing the BD-WASP or WASP^WH1^ mutant’s fusion proteins and the AD-WIP or empty vector. The transformed colonies were streaked on SD minimal media plates lacking Tryptophan/Leucine or Tryptophan/Leucine/Histidine and incubated at 30°C for 5 days. Additional file 2: Figure S2. WASP missense mutants are stably expressed in HEK293T cells. Expression of wild type WASP-RFP and WASP_R_-RFP (S1-WASP-shRNA resistant) in HEK293T cells (A) and Jurkat T cells (B) was analyzed by immunoblot using anti-WASP antibody. (C). WASP or its mutants tagged with GFP were expressed in HEK293T cells and analyzed for their expression using fluorescence microscope and by western blot (D) using anti-GFP antibody. Additional file 3: Figure S3. WASP mutants (WASP^L46P^ and WASP^A47D^) are stable in the presence of Hck. WASP_R_ or its mutants tagged with His tag were expressed in HEK293T cells together with either GFP or Hck-GFP. The cells were lysed and immunoblot analysis was carried with anti-His (WASP expression), anti-GFP (Hck or GFP) and GAPDH.
