# Supplementary figures and images for: X-Linked thrombocytopenia causing mutations in WASP (L46P and A47D) impair T cell chemotaxis
Source: J Biomed Sci. 2014 Sep 9;21(1):91. doi: 10.1186/s12929-014-0091-1 (PMC4266975; doi:10.1186/s12929-014-0091-1)

Figure S1

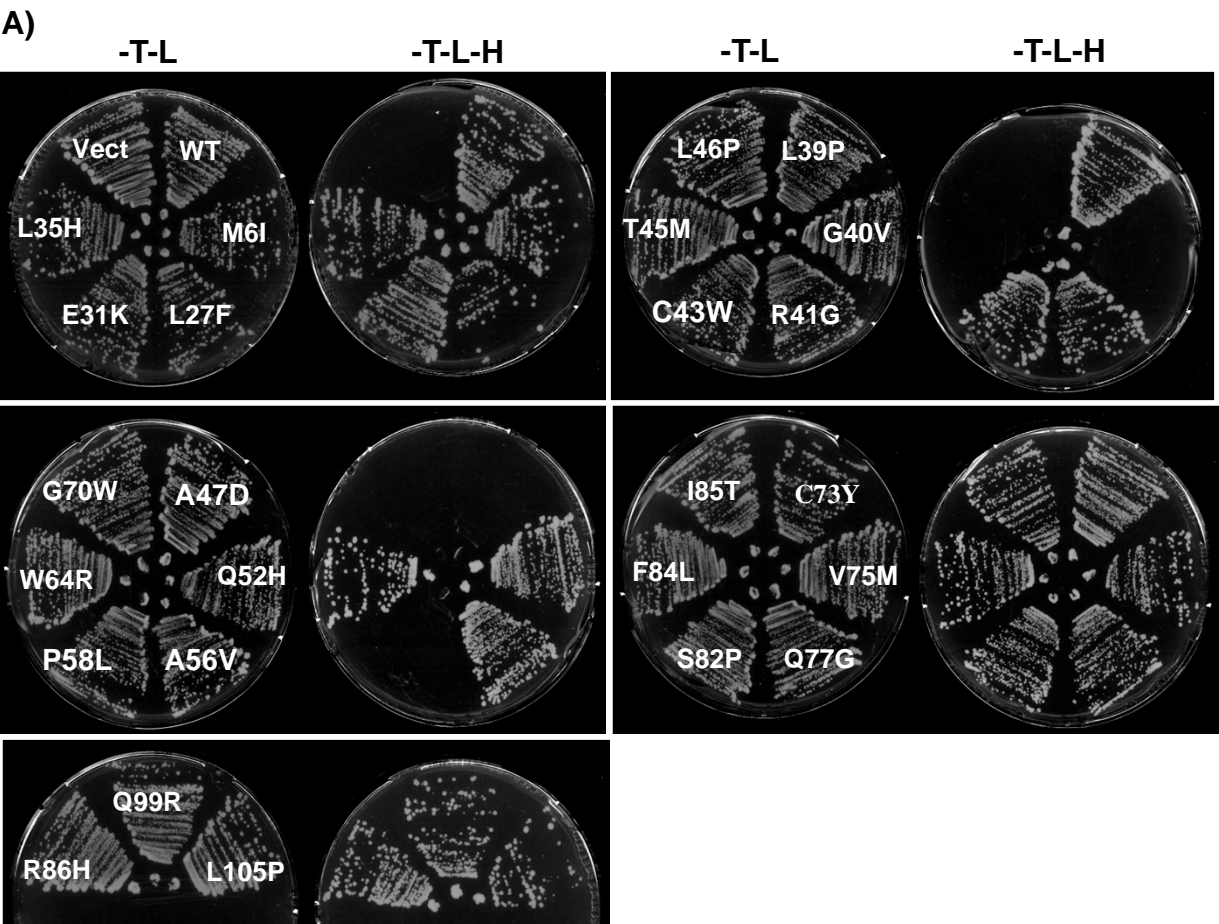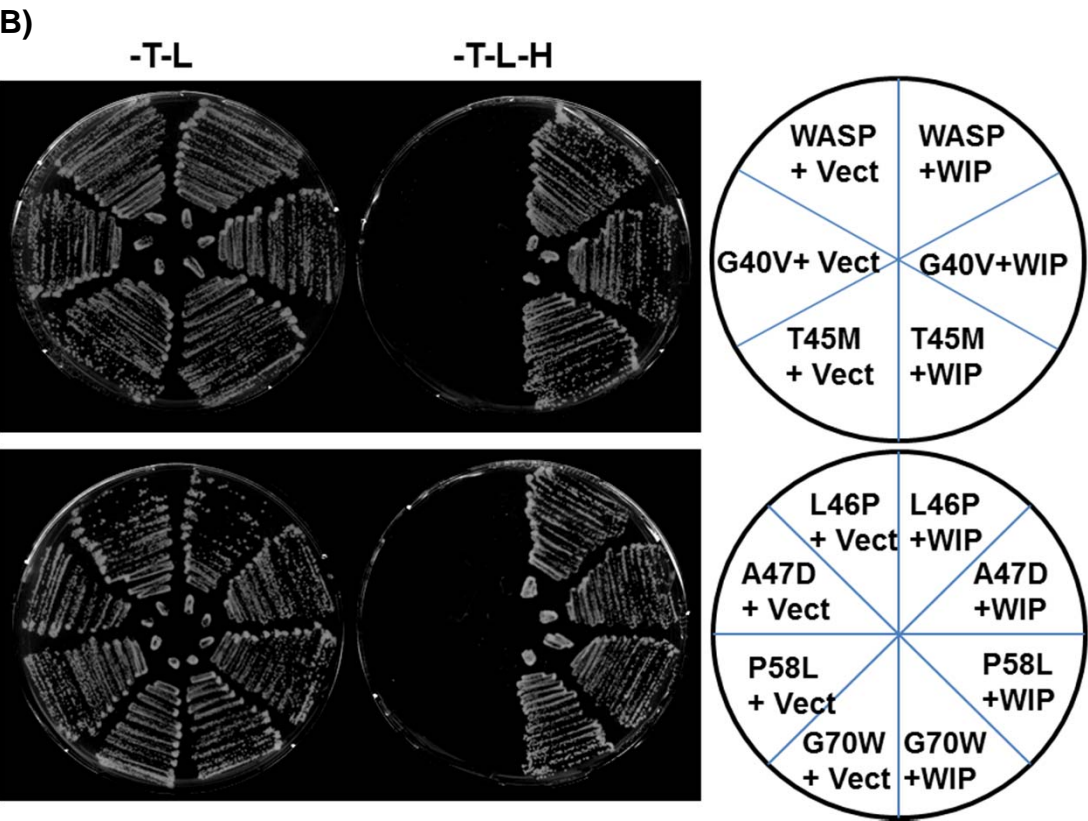

Supplement: Additional file 1: Figure S1. — Six missense mutations in the WH1 domain abolish WASP-CIB1 interaction. (A) Yeast two hybrid strains harboring Gal4 DNA-Binding domain-WASP1–265 (WT or its mutants) and Gal4 Activation Domain-CIB1 fusion were streaked on selective plates (−T-L: minus Tryptophan/Leucine or –T-L-H: minus Tryptophan/Leucine/Histidine). The plates were incubated at 30°C and photographed after 5 days. (B) Mutations affecting WASP-CIB1 interaction do not affect WASP-WIP complex formation. S. cerevisiae strain PJ69-4A was co-transformed with plasmids expressing the BD-WASP or WASPWH1 mutant’s fusion proteins and the AD-WIP or empty vector. The transformed colonies were streaked on SD minimal media plates lacking Tryptophan/Leucine or Tryptophan/Leucine/Histidine and incubated at 30°C for 5 days. [file 12929_2014_91_MOESM1_ESM.pdf]

**Figure S2**

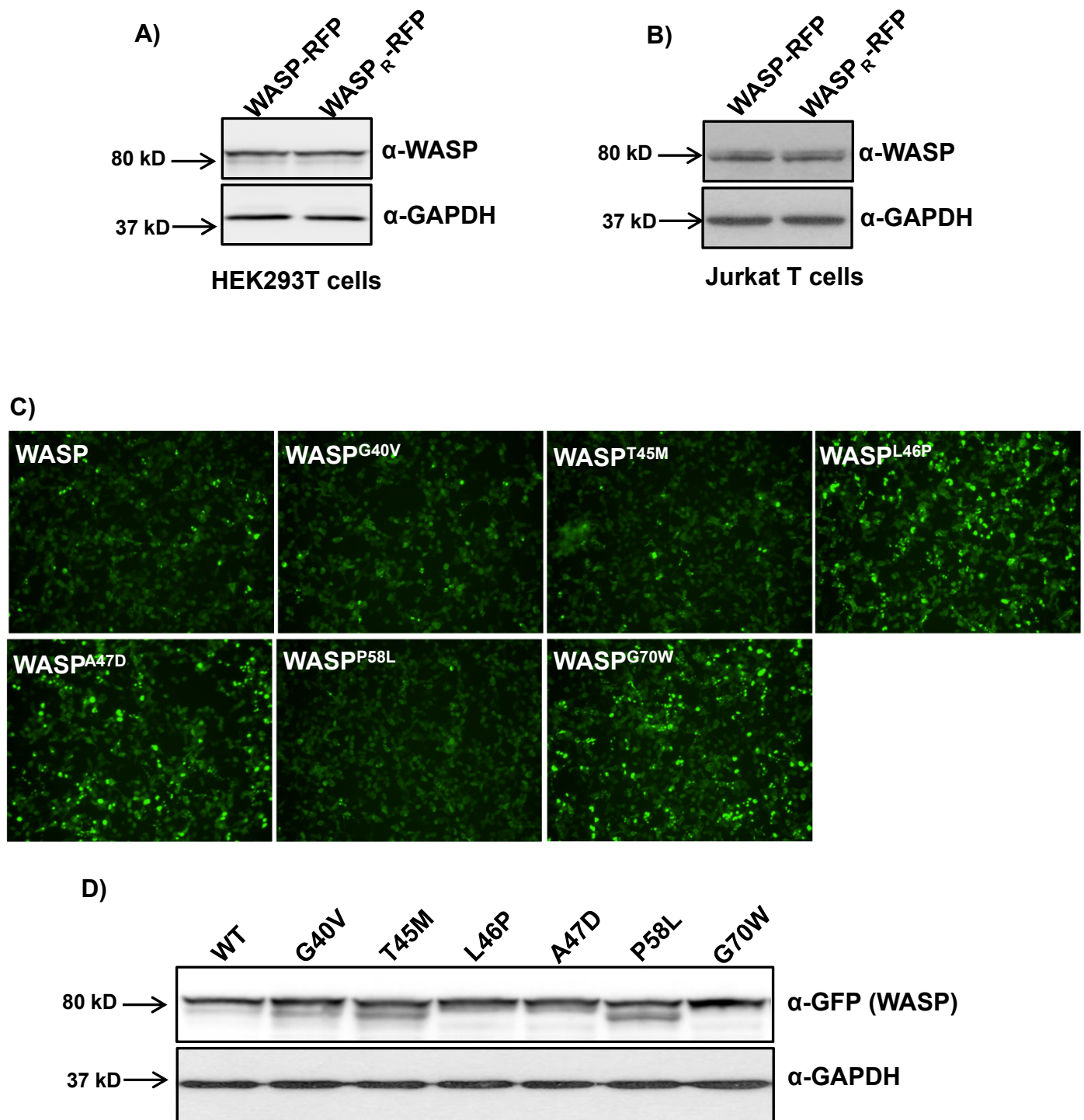

Supplement: Additional file 2: Figure S2. — WASP missense mutants are stably expressed in HEK293T cells. Expression of wild type WASP-RFP and WASPR-RFP (S1-WASP-shRNA resistant) in HEK293T cells (A) and Jurkat T cells (B) was analyzed by immunoblot using anti-WASP antibody. (C). WASP or its mutants tagged with GFP were expressed in HEK293T cells and analyzed for their expression using fluorescence microscope and by western blot (D) using anti-GFP antibody. [file 12929_2014_91_MOESM2_ESM.pdf]

Figure S3

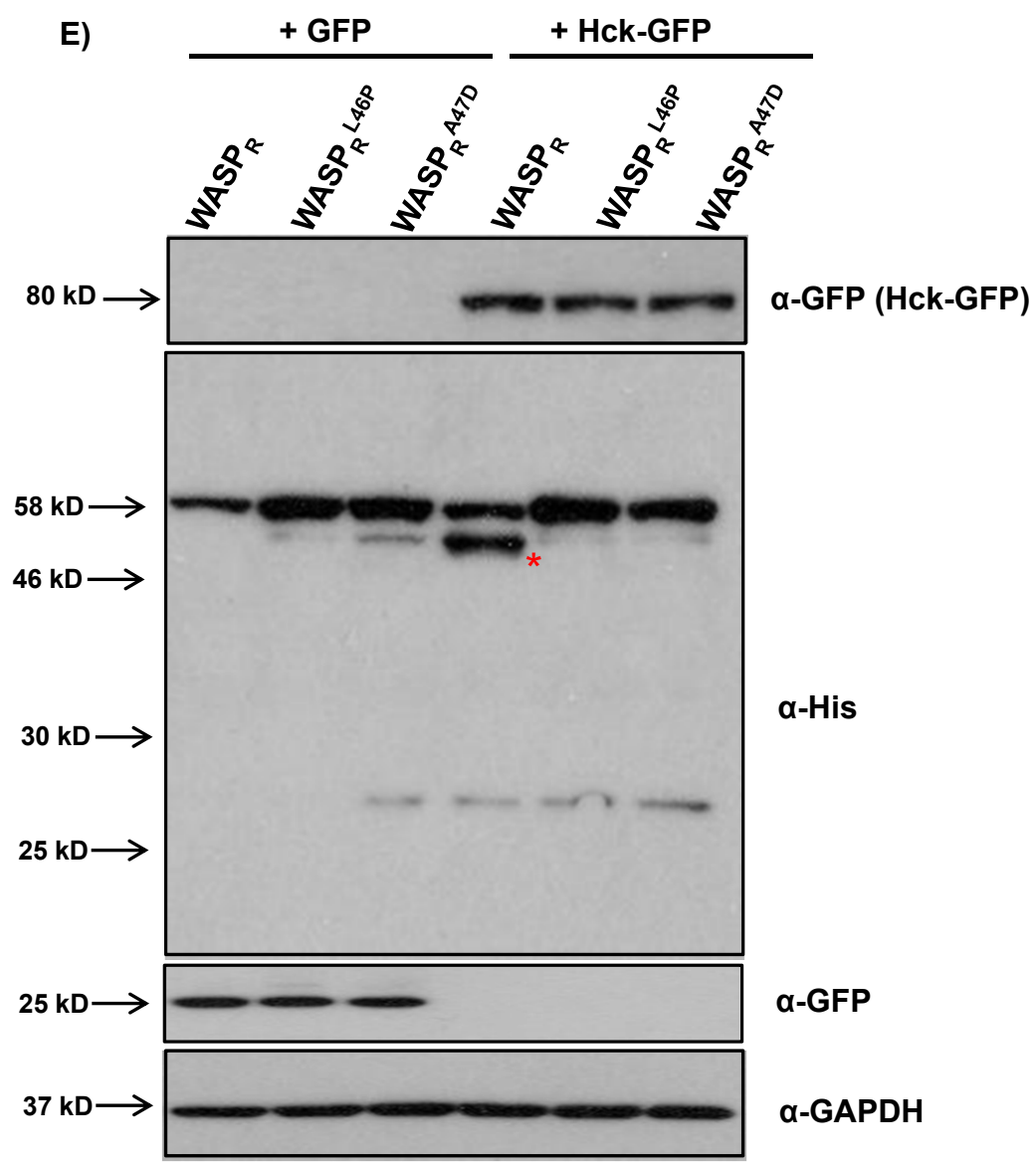

Supplement: Additional file 3: Figure S3. — WASP mutants (WASPL46P and WASPA47D) are stable in the presence of Hck. WASPR or its mutants tagged with His tag were expressed in HEK293T cells together with either GFP or Hck-GFP. The cells were lysed and immunoblot analysis was carried with anti-His (WASP expression), anti-GFP (Hck or GFP) and GAPDH. [file 12929_2014_91_MOESM3_ESM.pdf]
